# Effect of chitosan and curcumin nanoparticles against skeletal muscle fibrosis at early regenerative stage of glycerol-injured rat muscles

**DOI:** 10.1186/s12891-022-05633-x

**Published:** 2022-07-14

**Authors:** Mohamed A. A. Mahdy, Mohamed A. Akl, Fatma A. Madkour

**Affiliations:** 1 Department of Anatomy and Embryology, Faculty of Veterinary Medicine, South Valley University, Qena, 83523 Egypt; 2 Department of Pharmaceutics and Pharmaceutical Technology, Faculty of Pharmacy (Boys), Al-Azhar University, Nasr City, Cairo, Egypt

**Keywords:** Chitosan, Curcumin, Fibrosis, Glycerol injury, Nanoparticles

## Abstract

**Introduction:**

Chitosan and curcumin are natural products that have a wide range of beneficial effects including wound healing. However, their high molecular weight and poor water solubility limit their applications.

**Aims:**

Therefore, the current study aims to evaluate the effects of chitosan (Cs) and curcumin (Cn) nanoparticles (NPs) on fibrosis and regeneration of glycerol-injured muscle.

**Methods:**

Muscle injury was induced by intramuscular injection of glycerol into the tibialis anterior muscle of rats. Cs-NPs and Cn-NPs were administered at different doses intraperitoneally after injury. Injured muscles were collected at day 7 after injury, and muscle fibrosis and regeneration were assessed.

**Results:**

The present results revealed that Cs-NPs and Cn-NPs treatment significantly decreased fibrosis index and increased the average myotube diameter with shifting of the distribution of myotube diameters towards larger diameters in a dose-dependent manner. Immunohistochemical analysis revealed that Cs-NPs and Cn-NPs treatment significantly decreased the number of CD-68^+^ cells and Col-1^+^ area. Results showed that Cn-NPs had a higher protective effect, in the form of attenuating muscle fibrosis and inflammation, and enhancing muscle regeneration, than that of Cs-NPs.

**Conclusions:**

To our knowledge, this is the first study to document the effects of Cs-NPs in injured muscles. The results of study might be a novel approach to attenuate muscle fibrosis in humans using curcumin and chitosan nanoparticles.

## Introduction

Muscle fibrosis is a characteristic feature of muscular dystrophies [1], aging and following muscle injury [2]. It is developed due to the excessive accumulation of extracellular matrix (ECM) components, especially collagen in the endomysium and perimysium of skeletal muscle [2]. Muscle fibrosis is closely associated and overlapping with inflammation. It deteriorates the functional, as well as the structural properties of skeletal muscle [3, 4], impairs myofiber regeneration after injury [5], and increases muscle susceptibility to re-injury [6]. Furthermore, persistent fibrosis hinders both gene- and cell-based therapies used for the treatment of muscular dystrophies [7]. Therefore, it is essential to develop effective anti-fibrotic therapies as a strategy to enhance muscular dystrophies. Several factors have been used to ameliorate muscle fibrosis development due to their anti-fibrotic effect. These factors including TGF-β inhibitors, such as suramin and decorin, or myostatin inhibitors, such as follistatin [8].

We have shown that glycerol-injured rat muscle is a suitable model for studying muscle fibrosis. In this model, glycerol induces significant fibrosis at the early stage of regeneration in rat muscles which increases progressively together with persistent inflammatory cellular infiltration up to 2 weeks after injury [9]. In addition, TGF-β1 upregulates following glycerol injury and neutralization of TGF-β1 activity by a neutralizing antibody reduces muscle fibrosis and enhances muscle regeneration [10]. However, the beneficial effect of injecting a neutralizing antibody to TGF-β1 is local with no systematic beneficial effect. Therefore, it is urgent to find a suitable supplementation that reduces muscle fibrosis and improves muscle regeneration systematically.

Chitosan is a natural non-toxic, biocompatible, and biodegradable polysaccharide obtained by partial or complete deacetylation of chitin, the major structural component present in the exoskeletons of the crustaceans, shrimps, and crabs. Therefore, it has a wide biomedical application [11, 12]. Chitosan has been reported to accelerate wound healing through enhancing inflammatory cellular infiltration during the early phase of healing [13, 14], fibroblast proliferation, cytokine production, and ordered collagen deposition [15].

Curcumin is the main polyphenol present as an active ingredient in the Asian spice turmeric (*Curcuma longa*). Curcumin has a wide range of beneficial effects including anti-inflammatory and antioxidant effects [16, 17]. In vitro studies showed that curcumin suppresses the production of TNF-α and IL-1 by human macrophages [18]. In addition, curcumin ingestion reduces muscle damage following exercise [19], improves muscle performance, reduces muscle inflammation after hind limb ischemia [20] and following muscle injury [21–23]. Moreover, curcumin attenuates inflammation following exercise-induced muscle damage and ischemia-reperfusion injury [24, 25]. Systemic administration of curcumin improves muscle regeneration after trauma in a dose-dependent manner and this improvement occurs at very early times after injury [26].

Unfortunately, the high potential of chitosan and curcumin for treatment of chronic inflammation is hampered by poor water solubility and high molecular weight of curcumin are the major disadvantages that limit their applications. In addition, the degree of deacetylation affects the hydrophilicity and biocompatibility of chitosan polysaccharide [27]. To overcome these hurdles, nanoparticle formulation is used to increase the solubility, bioavailability, as well as therapeutic efficacy of the poorly soluble compounds via increasing their surface area [27–29].

To our knowledge, the effect of chitosan and curcumin nanoparticles on muscle fibrosis following injury in rats has not been studied yet. Therefore, the current study aims to evaluate the effects of chitosan and curcumin nanoparticles supplementation on fibrosis and regeneration during early phase of regeneration in glycerol-injured rat muscle.

## Materials and methods

### Reagents

Low molecular weight (LMW) chitosan (50-90 KDa, 75-85% deacetylation), curcumin from *Curcuma longa* (Tumeric) powder (C1386, mol wt: 368,38 g/mol), and Polyvinyl alcohol (PVA, mol wt: 31,000-50,000, 87-89% hydrolyzed) were purchased from Sigma-Aldrich (St. Louis, MO, USA). Poly (ε -caprolactone) and Penta-sodium tripolyphosphate (TPP) were purchased from Sigma-Aldrich (Tokyo, Japan). Glycerol was purchased from Advent Chembio (Mumbai, India), and picrosirius red staining kit was purchased from Polysciences (Eppelheim, Germany). The primary antibodies including anti-Collagen type 1 antibody (Col-1, EPR24331-53) was purchased from Abcam (Cambridge, UK) and anti-CD68 monoclonal antibody (FA-11) was purchased from Thermo Fisher Scientific (IL, USA).

### Preparation and characterization of nanoparticles

#### Preparation of chitosan nanoparticles (Cs-NPs)

Cs-NPs were prepared using the ionic gelation method based on cross linking between Cs solution and sodium tripolyphosphate (TPP) anions described early by Calvo, et al [30] Briefly, Cs was dissolved in 1% glacial acetic acid (2 mg/ml) using a magnetic stirring for 30 min and the pH was adjusted to 5.4 using triethanolamine. TPP 0.5% w/v was dissolved in distilled water. TPP aqueous solution (5 ml) was added to 10 ml of Cs solution dropwise at room temperature using magnetic stirring (at 1000 rpm, for 60 min) to form an opalescent suspension. Nanoparticles were collected by centrifugation at 12,000 rpm, 4 °C for 30 min and washed 3 times with deionized water using Sorvall RC-28S super speed centrifuge (Du Pont, Wilmington, DE, USA). Finally, the pellet of the nanoparticles was re-suspended in 5 ml of deionized water and stored at 4- 8 °C.

#### Preparation of curcumin (Cn) nanoparticles

Cn-Poly (ε-caprolactone) nanoparticles Cn-PCL NPs were prepared using the single emulsion-solvent evaporation technique described by Akl, et al [31] Briefly, 45 mg of PCL polymer was dissolved in 5 ml of chloroform: methanol (ratio 2:1) in a glass tube. Then 20 mg of curcumin powder was dissolved in the polymer/solvent mixture for 10 min with intermittent vortexing. The organic phase containing the polymer and curcumin was rapidly added dropwise into a glass tube containing 10 ml of 2% PVA in an aqueous solution, while vortexing. The tube contents were then emulsified by using sonication for 10 min at 70% amplitude in an ice-water bath by using an ultrasonic probe sonicator. The resulting fine (O/W) emulsion was immediately poured into 30 ml of an aqueous PVA 0.2% w/v solution under rapid stirring with a magnetic stirrer. The solvent was then evaporated from the droplets under magnetic stirring at 800 rpm for 5 h. The nanoparticles were collected by centrifugation at 20,000 rpm for 15 min and washed 3 times with deionized water using Sorvall RC-28S super speed centrifuge (Du Pont). Finally, the pellet of the nanoparticles was re-suspended in 5 ml of deionized water.

#### Characterization of the prepared nanoparticles

Determination of particle size and zeta potential

The mean particle size, the polydispersity index (PDI), and surface charge of the prepared Cn polymeric NPs or Cs-nanogel were determined with dynamic light scattering (DLS) using a Zetasizer Nano-ZS apparatus (Malvern Panalytical Ltd., Worcestershire, UK). Briefly, 300 μl of nanoparticle suspension was diluted with 3 ml of Milli-Q water and sonicated for 30 s. Measurements were carried out at room temperature with a detection angle of 90^о^. Each sample was measured in triplicate and the obtained results were expressed as mean ± SD.

Fourier transform infrared spectroscopy (FT-IR)

Fourier transform infrared spectroscopy (FT-IR) was done to evaluate the spectrum of TPP powder, Cs-TPP NPs, PCL powder, and Cn-PCL NPs using a Nicolet 380 spectrophotometer (Thermo Fisher Scientific, MA, USA) in 400–4000 cm^− 1^ region. The average of characteristic peaks of IR transmission spectra was recorded from triplicate samples.

Morphology of the formulated nanoparticles

Morphological analysis of the formulated nanoparticles was assessed using a transmission electron microscope (TEM). A droplet of freshly prepared nanoparticle suspensions in aqueous media was placed onto a copper grid supported with a carbon film. The excess solution was absorbed with a filter paper. Grids were examined directly with JEOL100CX II (JEOL, Tokyo, Japan) at accelerating voltage of 80 kV without further staining.

### Muscle injury and nanoparticles treatment

#### Animals and ethical approval

Adult male Wistar rats (8 weeks-old, 180-200 g body weight) were housed in plastic cages in a thermally controlled room (23 ± 1 °C) with 12 h light-dark cycle with free access to food (standard chow diet) and water throughout the experiment. Animals were acclimatized for 1 week before the experiment. Animal experiments were carried out in accordance with the ARRIVE guidelines and following the guidelines and regulations of the Animal Ethics Committee for Veterinary Research, Faculty of Veterinary Medicine, South Valley University, Qena, Egypt. The experimental protocol was approved by the Animal Ethics Committee for Veterinary Research, Faculty of Veterinary Medicine, South Valley University, Qena, Egypt (approval number: 3a-09-2020).

#### Study design

Muscle injury was induced by intramuscular injection of glycerol according to the method reported by Mahdy, et al. [9] as follows: rats were anesthetized by intraperitoneal injection of 10 mg/kg of 2% xylazine using 2 ml plastic syringe with 23G needle, and the left hind limb was shaved and disinfected, then the tibialis anterior (TA) muscle was injected with 500 μl of glycerol 50% (*v/v*) in sterile phosphate-buffered saline (PBS), pH 7.4 using insulin syringe. Animals were then randomly divided into nine experimental groups (*n* = 8/group) as follows: “control group”, animals received glycerol only with no nanoparticle’s treatment. Chitosan nanoparticles-treated groups (4 treated groups received different doses of Cs-NPs): Cs-50, Cs-150, Cs-200, and Cs-400 in which rats received 50, 150, 200, and 400 μg of Cs-NPs, respectively. Curcumin nanoparticles-treated groups (4 treated groups received different doses of Cn-NPs): Cn-5, Cn-15, Cn-20, and Cn-40 in which rats received 5, 15, 20, and 40 μg of Cn-NPs, respectively. For the single dose of NPs, 1 ml PBS containing either Cs-NPs or Cn-NPs was injected intraperitoneally (IP) 1 h after muscle injury. For the doses, Cs-400 and Cn-40, rats received two doses of Cs-NPs (200 μg each) and Cn-NPs (20 μg each), respectively. The first dose was injected 1 h after injury and the second dose was injected at day 4 after muscle injury. All animals were sacrificed 7 days after glycerol injury based on our previous results showing that glycerol induces significant fibrosis at day 7 after injury (fibrosis index is 29.8 ± 3.9%) [9].

#### Histological analysis

Animals were anaesthetized by an overdose of anesthesia (20 mg/kg xylazine) in a closed chamber. Muscle samples were dissected and then fixed in 10% neutral buffered formalin (NBF). Fixed samples were processed for paraffin embedding staining. Sections at 5 μm thickness were cut and stained with Hematoxylin and Eosin (HE) staining for morphological analysis. Collagen fibers were stained with picrosirius red staining kit according to the manufacturer’s protocol. Stained sections were examined using Leica DMLS microscope (Leica Microsystems, Wetzlar, Germany) and digital images were obtained by Leica ICC50 HD camera attached to the microscope.

#### Morphometric analysis

Morphometric measurements were done by an independent researcher who was unaware of the experiment. Quantification of muscle fibrosis was done by measuring the Sirius red-positive area according to the method reported by Mahdy, et al [9] Three to five non-overlapping fields at a magnification of 100×, were analyzed per section and three sections per animal were selected. Fibrosis index was calculated as the ratio of the Sirius red-positive area to the total muscle area in each field after adjusting the threshold using Fiji Image-J 1.53c software (National Institutes of Health, Bethesda, MD, USA). Morphometric analysis of muscle regeneration was carried out on HE-stained images. The minimum Feret’s diameters (smallest diameters) of about 150 randomly selected regenerated myotubes (with central nuclei) were measured using the Fiji Image-J 1.53c software. The mean ± SD was calculated for each group and histograms showing the distribution of myotube diameters were presented [10].

#### Immunohistochemistry

Muscle sections were deparaffinized, subjected to antigen retrieval using EDTA solution (pH 8), and they were then treated with hydrogen peroxide 0.3% and protein block, followed by incubation with rabbit monoclonal anti-Collagen type 1 antibody (1: 800 dilution) and anti-CD68 monoclonal antibody (1: 100 dilution). The slides were rinsed three times with PBS, incubated with anti-mouse IgG secondary antibodies (EnVision+System-HRP; Dako, CA, USA) for 30 min at room temperature, visualized with di-aminobenzidine commercial kits (Liquid DAB+Substrate Chromogen System; Dako), and finally counterstained with Mayer’s hematoxylin. As a negative control procedure, the primary antibodies were replaced by normal serum. The CD-68^+^cells/field were counted and Col-1^+^ area/field was measured using Fiji Image-J 1.53c software in about 8 to10 high power fields [32, 33].

#### Statistical analysis

Data were analyzed with SPSS software, version 21 (IBM SPSS, Chicago, IL, USA). One-way ANOVA followed by Dunnett’s *post-hoc* test was used to compare the treated groups with the glycerol-injured group, and Bonferroni’s *post-hoc* test was used to compare between treatments. Data were presented as mean ± SD, and the significant difference was set as *P* < 0.05.

## Results

### Nanoparticle’s characterization

#### Zeta sizer and zeta potential

**Fig. 1:**
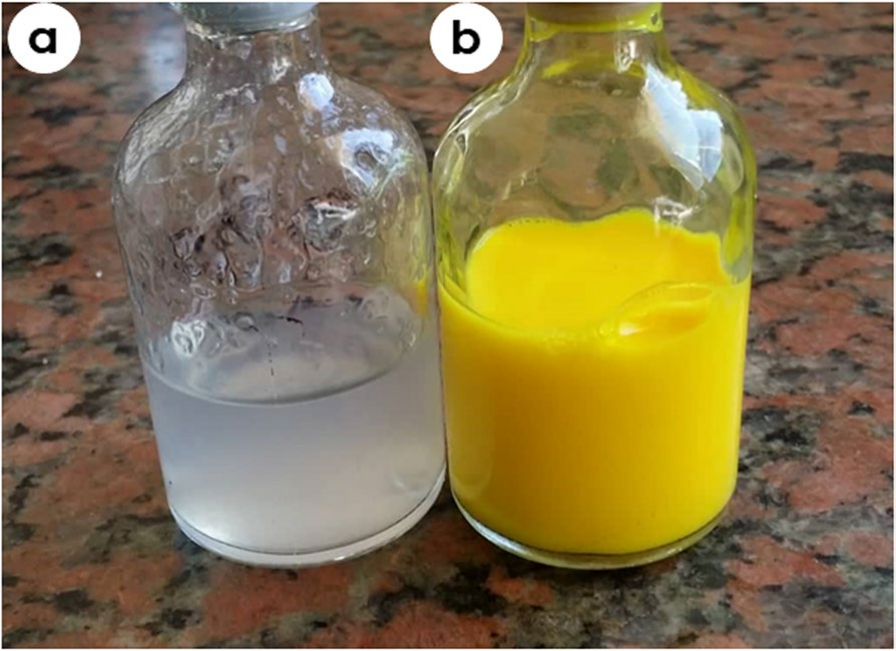
A photograph showing the macroscopic appearance of (**a**) chitosan-TPP (whitish color) and (**b**) curcumin-PCL (yellowish color) nanogel solutions

**Fig. 2:**
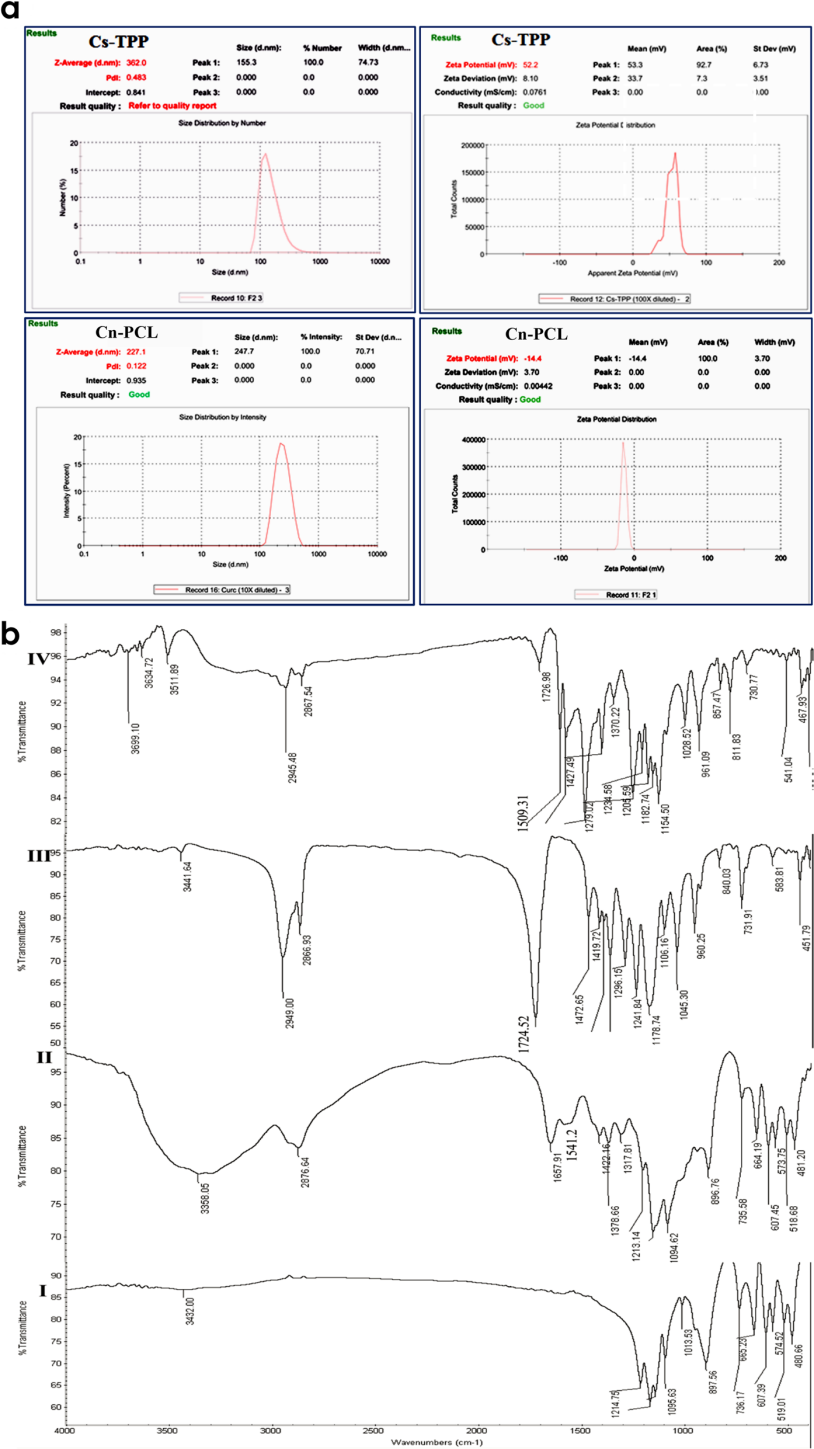
**a** Analysis of particle size and zeta potential of chitosan-TPP (Cs-TPP) and curcumin-PCL (Cn-PCL) nanoformulations. **b** FTIR analysis of the TPP powder (I), Cs-TPP NPs (II), PCL powder (III), and Cn-PCL NPs (IV)

The ionotropic gelation and single-emulsion ultrasonication methods were successfully utilized for preparing CS-TPP and Cn-PCL nanogel formulations, respectively (Fig. 1a, b). The formulations were evaluated for particle size, PDI, and zeta potential (ZP). The mean particle size was in the nanometer range (362 ± 3.02 and 227.1 ± 1.42 nm for Cs-NPs and Cn-PCL NPs, respectively), and both exhibited very small PDI values (i.e., between 0.317 ± 0.094 and 0.122 ± 0.011 for Cs-NPs and Cn-PCL NPs, respectively), which showed that the particle size populations of the prepared Cs-TPP and Cn-PCNPs nanoparticles were very homogeneous. A small PDI value (i.e., ~ 0.2) is often taken as an indication of a homogenous particle size population. The ZP of Cs-TPP formulation exhibited positively charged value of 52.2 mV, while the Cn-PCNPs formulation displayed a high negative value of − 14.4 mV (Fig. 2a).

#### FTIR results

Chemical changes in the NP formulation were evaluated by FTIR analysis. The characteristic spectrum of TPP powder, Cs-TPP NPs, PCL powder, and Cn-PCL NPs are shown in Fig. 2b. The FTIR spectrum of TPP (Line I), revealed absorption characteristic bands for P=O stretching at 1214.75 cm^− 1^, 1148 cm^− 1^ for symmetric and antisymmetric stretching vibrations in PO2 group, small peak for symmetric and antisymmetric stretching vibrations in PO3 group at 1095.63 cm^− 1^, and 897.56 cm^− 1^ for antisymmetric stretching of the P-O-P bridge.

In Cs-TPP NPs (line II) showed the basic characteristic absorption band of CS and TPP but shift to lower frequencies, in which the tip of the peak of 3432 cm^− 1^ has a shift to 3358.05 cm^− 1^ and becomes wider with increased relative intensity indicating an enhancement of hydrogen bonding. Also, the peaks of N-H bending vibration of amide І at 1600 cm^− 1^ and the amide ІІ carbonyl stretch at 1650 cm^− 1^ shifted in nanoparticles formulation to 1541.2 cm^− 1^ and 1657.91 cm^− 1^, respectively. Additionally, CS-TPP cross-linked showed peak at 1213.14 cm^− 1^ for P=O.

For PCL (line III), the characteristic band at 3441.64 cm^−^ was attributed to the terminal OH group. A peak at 2949 and 2866.93 cm^− 1^ was attributed to the asymmetrical and symmetrical C − H2 stretching, the strong bands appearing at 1724.52 cm^− 1^ were assigned to the carbonyl stretching and several sharp peaks 1296. 15 cm^− 1^, 1241.84 cm^− 1^, 1178.74 corresponding to C–O and C–C stretching in the crystalline phase, asymmetric COC, and Symmetric COC stretching, respectively. As expected, the FT-IR spectrum of Cn-PCL nanoparticles (line IV) showed the basic characteristic peaks of PCL, and curcumin without any significant change, but shift to higher frequencies at 1509.31 and 1427.79 cm^− 1^ which attributed to the stretching vibrations of C–C of the benzene ring and olefinic bending vibration of the C=C group bound to the benzene ring, respectively, which confirms the encapsulation of curcumin by the PCL polymer (Fig. 2b).

#### Morphology of the nanoparticle formulations

**Fig. 3:**
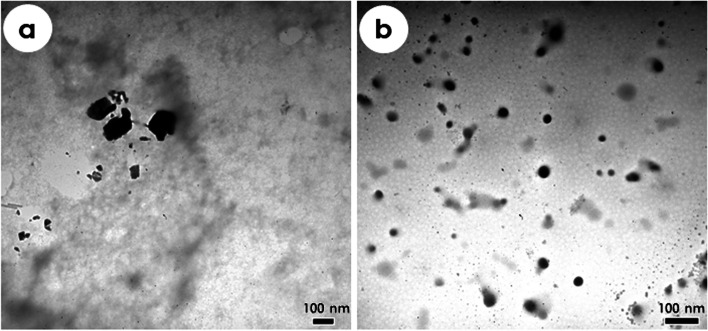
TEM micrographs of the formulated Cs-TPP (**a**) and Cn-PCL (**b**) nanoparticles

TEM images revealed nanostructures of the formulated nanoparticles with a homogeneous size distribution. TEM images showed that both Cs-NPs and Cn-NPs presented nanostructures with a spherical morphology with no evidence of particle aggregation (Fig. 3a, b).

### Treatment with chitosan and curcumin nanoparticles ameliorated fibrosis and enhanced regeneration in glycerol-injured rat muscles

#### Chitosan and curcumin nanoparticles ameliorated muscle fibrosis

**Fig. 4:**
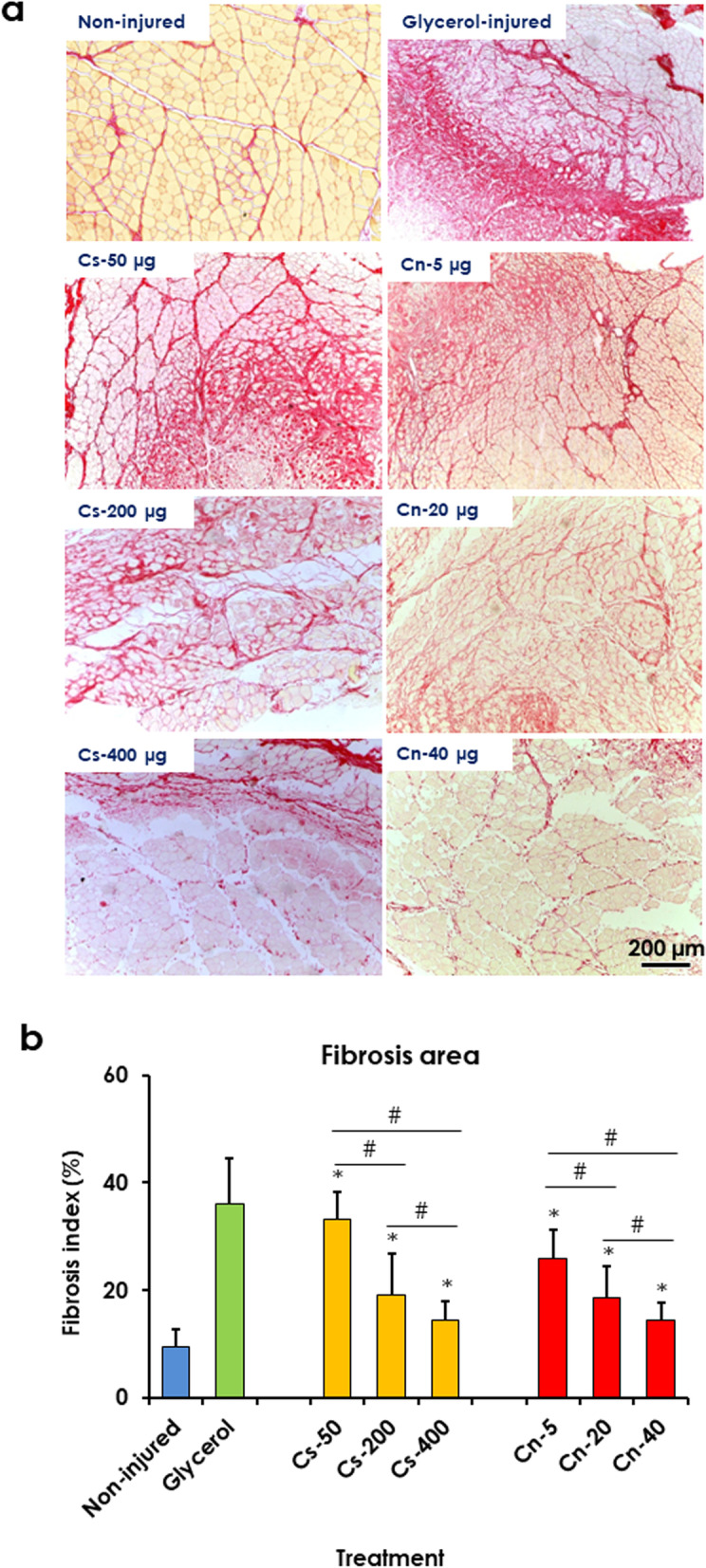
Treatment with chitosan (Cs) and curcumin (Cn) NPs decreased fibrosis index in glycerol-injured rat muscles. **a** Light micrographs of rat tibialis anterior (TA) muscles from non-injured muscle (negative control), glycerol-injured (positive control), muscle treated with Cs-50, − 200, and − 400 μg, and Cn-5, − 20, − 40 μg. Sirius red stain. **b** Quantification of Sirius red-positive area. Data are expressed as means ± SD, * indicates significant difference from glycerol-injured muscle, # indicates significant difference between treatment groups (*P* < 0.05)

Measurements of the Sirius red-positive area revealed that Cs-NPs treatment significantly decreased fibrosis index by about 7.82, 46.82, and 59.76% in treated rats with 50, 200, and 400 μg, respectively than that in the control rat muscle. While Cn-NPs treatment significantly decreased fibrosis index by about 28.14, 48.55, and 59.97% in treated rats with 5, 20, and 40 μg, respectively than that in the control rat muscle. These results indicated that the fibrosis index was decreased following treatment with Cs-NPs and Cn-NPs in a dose-dependent manner (Fig. 4).

#### Chitosan and curcumin nanoparticles enhanced muscle regeneration

**Fig. 5:**
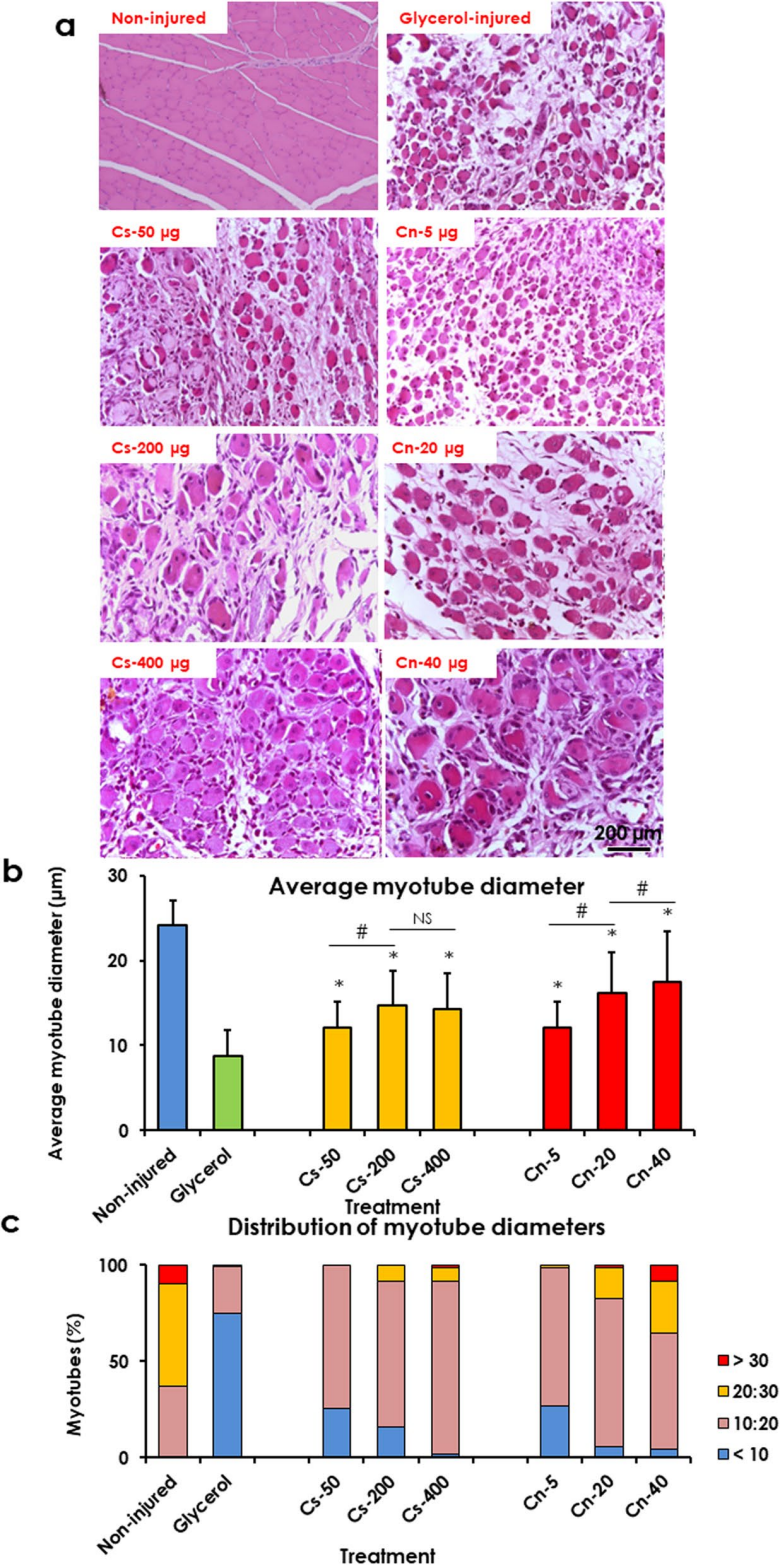
Treatment with chitosan (Cs) and curcumin (Cn) NPs enhanced muscle regeneration in glycerol-injured rat muscles. **a** Light micrographs of rat tibialis anterior (TA) muscles from non-injured muscle (negative control), glycerol-injured (positive control), muscle treated with Cs-50, − 200, and − 400 μg, and Cn-5, − 20, − 40 μg. HE stain. **b** Treatment with Cs and Cn NPs increased the average myotube diameters. **c** Treatment with Cs and Cn NPs shifted the distribution of myotube diameters towards higher values. Data are expressed as means ± SD, NS, not significant, * indicates significant difference from glycerol-injured muscle, # indicates significant difference between treatment groups and significant difference is indicated (*P* < 0.05)

Analysis of the average diameters of the regenerated myotubes revealed that Cs-NPs treatment increased average myotube diameter by about 1.38-fold, 1.64-fold, and 1.68-fold higher than that in the control rat muscle in treated rats with 50, 200, and 400 μg, respectively. While Cn-NPs treatment increased average myotube diameter by about 1.38-fold, 1.85-fold, and 2.0-folds higher than that in the control rat muscle in treated rats with 5, 20, and 40 μg, respectively (Fig. 5a, b). Analysis of the myotube diameters distribution revealed that both Cs-NPs and Cn-NPs treatment shifted the myotube diameters distribution towards larger diameters, than those in the control group. The number of myotubes with small diameters (less than 10 μm) decreased by about 66, 78, and 97% in rats treated with 50, 200, and 400 μg of Cs-NPs, respectively. While the number of myotubes with small diameters decreased by about 64, 92 and 94% in rats treated with 5, 20, and 40 μg of Cn-NPs, respectively. Furthermore, treatment with 40 μg of Cn-NPs increased the number of myotubes with larger diameters (more than 20 μm) by about 2.05-fold than that in 20 μg of Cn-NPs (Fig. 5c).

#### Chitosan and curcumin nanoparticles reduced inflammation and collagen deposition in glycerol-injured rat muscles

**Fig. 6:**
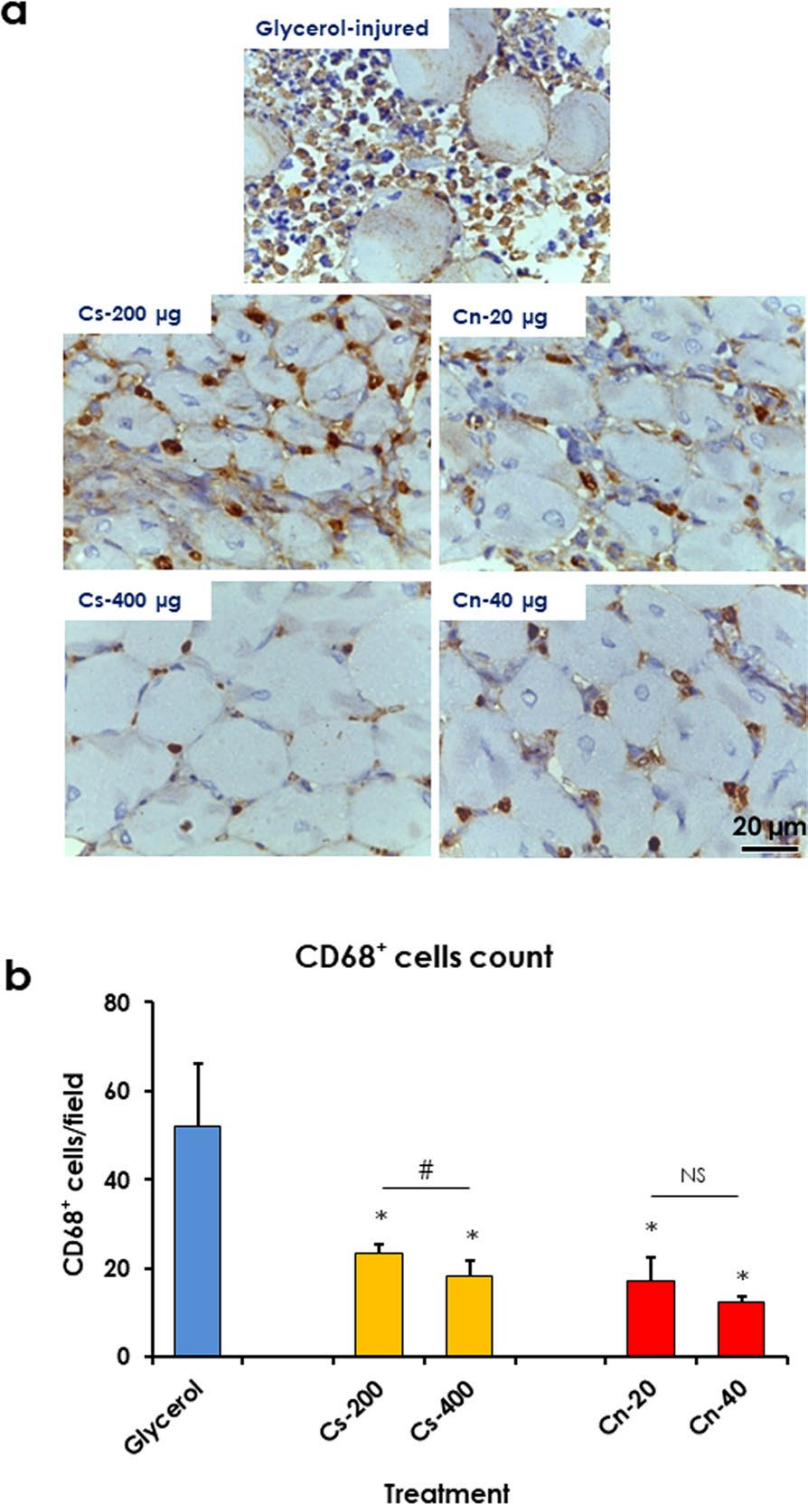
Treatment with chitosan (Cs) and curcumin (Cn) NPs decreased infiltrated macrophages in glycerol-injured rat muscles. **a** Light micrographs of muscles from control (glycerol-injured), treated with Cs-50, − 200, and − 400 μg, and Cn-5, − 20, − 40 μg. labelled with anti-CD-68 antibody. **b** Treatment with Cs and Cn NPs significantly decreased the CD-68^+^ cells. Data are expressed as means ± SD, NS, not significant, * indicates significant difference from glycerol-injured muscle, # indicates significant difference between treatment groups and significant difference is indicated (*P* < 0.05)

**Fig. 7:**
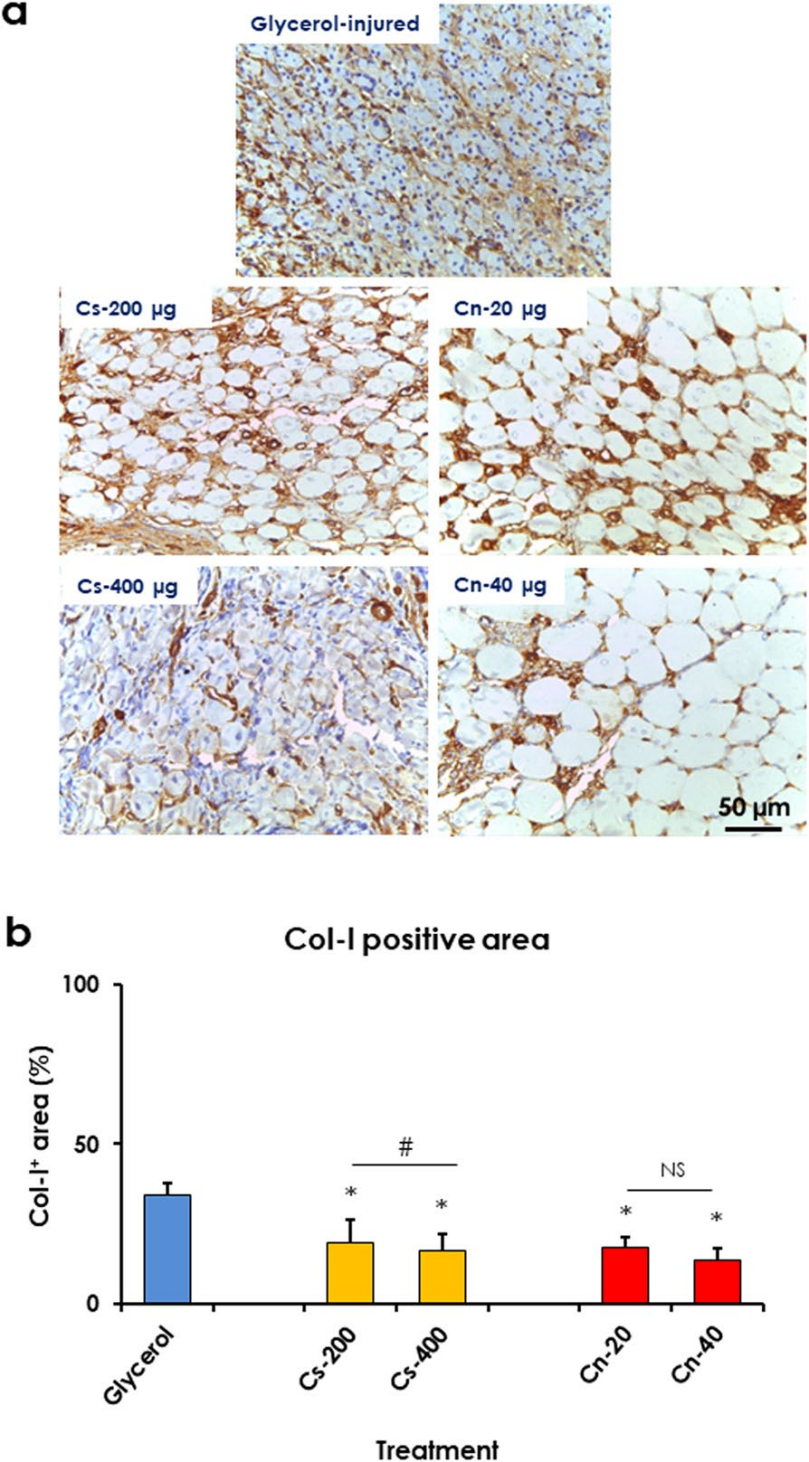
Treatment with chitosan (Cs) and curcumin (Cn) NPs decreased Col-1^+^ area in glycerol-injured rat muscles. **a** Light micrographs of muscles from control (glycerol-injured), treated with Cs-50, − 200, and − 400 μg, and Cn-5, − 20, − 40 μg. labelled with anti-Col-1 antibody. **b** Treatment with Cs and Cn NPs significantly decreased the Col-1^+^ area. Data are expressed as means ± SD, NS, not significant, * indicates significant difference from glycerol-injured muscle, # indicates significant difference between treatment groups and significant difference is indicated (*P* < 0.05)

Muscle sections were stained with the macrophage (anti-CD-68) and collagen type 1 (anti-Col-1) markers, and the number of CD-68^+^ cells was counted in each field. In addition, the Col-1a^+^ area was measured in each field. Treatment with 200 and 400 μg of Cs-NPs significantly decreased the number of CD-68^+^ cells by about 55 and 65%, respectively. While treatment with 20 and 40 μg of Cn-NPs significantly decreased the number of CD-68^+^ cells/field by about 67 and 76%, respectively (Fig. 6). On the other hand, treatment with 200 and 400 μg of Cs-NPs significantly decreased Col-1^+^ area by about 44 and 51%, respectively. While treatment with 20 and 40 μg of Cn-NPs significantly decreased Col-1^+^ area by about 49 and 61%, respectively (Fig. 7).

## Discussion

The present study evaluated the effects of Cs-NPs and Cn-NPs treatment on fibrosis and regeneration of glycerol-injured muscle. The present results revealed that treatment with Cs-NPs and Cn-NPs significantly decreased fibrosis index enhanced muscle regeneration, and decreased inflammation and collagen deposition in glycerol injured rat muscles in a dose-dependent manner.

In the current study, Cs of LMW was used to prepare Cs-NPs using the ionic gelation method. Cs of LMW produces the smallest NPs compared to medium and high molecular weight chitosan [34]. Smaller size NPs can easily penetrate the tissues [35]. In addition, the ionotropic gelation method is an easy and rapid method to synthesize Cs-NPs in which particle size can be controlled by changing the Cs to TPP ratio, molecular weight of Cs, and the pH of the reaction [36]. TPP is the favorable anionic cross-linker for chitosan because of its biocompatibility, biodegradability, and nontoxic nature [12, 37].

On the other hand, Cn-PCL NPs were prepared using single emulsion-solvent evaporation technique. PCL is a biodegradable biopolymer that is characterized by high permeability and excellent biocompatibility. Therefore, it is widely used for controlled drug delivery [38, 39]. Its low molecular weight particles undergo complete intracellular digestion within 14 days [39]. PCL polymer has been reported as an appropriate nanocarrier for lipophilic curcumin to overcome the low bioavailability and hydrophobic characteristic of curcumin [40, 41].

The particle size and DPI of NPs are the main physicochemical characteristics that affect the cellular uptake of NPs [42]. The present results showed that the mean particle size of Cn-PCL NPs was 227.1 ± 1.42 nm with very small PDI value (0.101 ± 0.011). These results are consistent with the particle size and PDI reported previously for curcumin encapsulated in PCL polymer [41, 43]. A small PDI value indicates a homogenous particle size population [43].

The Cs-TPP formulation resulted in a positively charged ZP of 52.2 mV. The positive charge of Cs-NPS facilitates its adherence to the negatively charged cell membrane, prolongs the drug release time, and enhances the drug’s availability to the internal tissues [44]. While Cn-PCNPs formulation displayed a high negative value of − 14.4 mV because of the carboxylic end groups of the PCL polymer [43]. The electronegativity of Cn-PNPs suggests effective cellular uptake of NPs [45]. It has been reported that synthesized NPs with ZP above ±30 mV are expected to be stable in suspension for longer time with no particles aggregation [34]. Taken together, a ZP value for nanoparticles in this range usually expects that the nanoparticles were well dispersed in the aqueous solution and that the nanosuspensions would have very good stability and tolerance against aggregation.

After Cs-TPP NPs crosslinking, the band at 1600 cm^− 1^ shifted to 1541 cm^− 1^and the band at 1650 cm^− 1^ shifted to 1657 cm^− 1^. These results have been attributed to the linkage between phosphoric and ammonium ions [46]. So, it is concluded that the tripolyphosphoric groups of TPP were linked with ammonium groups of chitosan. The inter- and intra-molecular actions were enhanced in chitosan nanoparticles. For Cn-PCL NPs there was a shift to higher frequencies at 1509.31 and 1427.79 cm^− 1^ confirming the encapsulation of curcumin by the PCL polymer [31].

The glycerol-induced muscle injury model has been reported as a good model to induce muscle fibrosis in rat. It is characterized by significant muscle fibrosis at the early stage of regeneration (day 7) which increases progressively up to 2 weeks after injury. Moreover, persistent inflammatory cellular infiltration is detected up to day 14 after injury [9] resulting in impaired muscle regeneration [9, 47]. The persistence of inflammatory cells alters the extracellular environment, increases the secretion of various inflammatory cytokines that contribute to muscle fibrosis [48]. The current morphometric analysis showed that treatment with chitosan and curcumin nanoparticles significantly reduced fibrosis in glycerol-injured rat muscles which was evidenced by the significant reduction of Sirius red-positive area and collagen type 1-positive area. The present results are in line with that of Ezhilararasan, et al. [49] who reported an innate affinity of chitosan NPs for ECM proteins, such as collagen. Administration of nano chitosan [50] and nano curcumin-chitosan mixture [51] reduce collagen fibers and inflammatory cells following tetrachloride-induced hepatotoxicity. These results further support the present finding showing a significant reduction in the number of CD-68^+^ cells following treatment with chitosan and curcumin nanoparticles. In addition, nano curcumin supplementation significantly decreased muscle fibrosis following surgical muscle laceration injury [52].

The present morphometric measurements showed that the treatment with chitosan and curcumin nanoparticles significantly enhanced muscle regeneration which was evidenced by the increased average myotube diameter and shifting in the distribution of myotube diameters towards larger values. This result agrees with the previous result showing significantly higher muscle fibers regeneration in nano curcumin-supplemented animals than the control group following surgical muscle laceration injury [52].

Previous studies revealed upregulation of TGF-β1 following glycerol injury [10]. TGF-β increases membrane fragility of myofibers through enhancement of oxidative stress and ROS activity within skeletal muscle fibers resulting in greater myofiber necrosis and reduces satellite cell activation [53]. In addition, TGF-β1 activates the expression of genes encoding ECM proteins, such as fibronectin and collagen and decrease ECM degrading proteases, such as matrix metalloproteinases resulting in muscle fibrosis [4]. Inhibition of TGF-β1 activity reduces muscle fibrosis and enhances muscle regeneration [10, 54, 55]. It has been reported that chitosan administration ameliorates pulmonary fibrosis in rats through inhibition of the proinflammatory cytokines including TGF-β1, and TNF-α [56]. Furthermore, curcumin inhibits fibrosis through inhibition of TGF-β1 signaling in and decreases ECM production [57, 58]. Taken together, it is suggested that the ameliorating effect of Cs and Cn NPs against muscle fibrosis might be through the inhibition of TGF-β1 signaling. Further molecular studies are recommended to confirm this hypothesis.

## Conclusions

In conclusion, the present study revealed that treatment with chitosan and curcumin nanoparticles significantly decreased muscle fibrosis, enhanced muscle regeneration, and decreased inflammation and collagen deposition at early regenerative stage in glycerol injured rat muscles. To the best of our knowledge, this is the first study to document the beneficial effects of chitosan nanoparticles in injured muscles. Further molecular studies are recommended to elucidate the possible mechanism of action of nanoparticles in glycerol-injured rat muscles.

### Limitations of study

The present study has some limitations. The effect of nanoparticles treatment has been evaluated at early regenerative stage (Day 7) following glycerol injury. However, extending the experiment up to day 21 after injury might provide complete picture of the fibrosis/ regeneration process. The present experiment evaluated M1 macrophage content by CD68 staining which does not provide overall evaluation of the entire inflammatory response. Moreover, the present study did not evaluate the functional status of the muscle which should be done in the future research. Treatment with nanoparticles should be compared with the approved drugs for muscle fibrosis.

## Data Availability

The datasets generated and/or analysed during the current study are not publicly available due [home institution does not have agreement for public data sharing] but are available from the corresponding author on reasonable request.

## Abbreviations

Col-1: Collagen type 1 antibody
Cn: Curcumin
Cs: Chitosan
ECM: Extracellular matrix
HE: Hematoxylin and Eosin
FT-IR: Fourier transform infrared spectroscopy
LMW: Low molecular weight
NPs: Nanoparticles
PBS: Phosphate-buffered saline
PCL: Poly (ε-caprolactone)
PDI: polydispersity index
PVA: Polyvinyl alcohol
SD: Standard deviation
TA: Tibialis anterior
TEM: Transmission electron microscope
TPP: Tripolyphosphate
ZP: Zeta potential
